# Meeting report: South African Medical Research Council Standard of Care in Clinical Research in Low- And Middle-Income Settings Summit, November 2017

**DOI:** 10.1186/s13063-021-05754-z

**Published:** 2021-11-06

**Authors:** Maurine D. Miner, Linda-Gail Bekker, Tamara Kredo, Niresh Bhagwandin, Lawrence Corey, Glenda E. Gray

**Affiliations:** 1 HVTN, Vaccine and Infectious Disease Division, Fred Hutchinson Cancer Research Center, 1100 Fairview Avenue North, Mail-stop E3-300, Seattle, WA 98109 USA; 2 Desmond Tutu HIV Centre, University of Cape Town, P.O. Box 13801, Mowbray, Cape Town, 7705 South Africa; 3 South African Medical Research Council, Francie van Zijl Drive, Parowvallei, Cape Town, PO Box 19070, Tygerberg, 7505 South Africa; 4 Division of Clinical Pharmacology, Department of Medicine, Faculty of Medicine and Health Sciences, Stellenbosch University, Private Bag X1, Matieland, Stellenbosch, 7602 South Africa; 5 Department of Medicine and Laboratory Medicine, University of Washington, 1959 NE Pacific St, Seattle, WA 98195 USA

**Keywords:** Pre-exposure prophylaxis, South Africa, Standard of care, LMIC, HIV/AIDS, Clinical trials

## Abstract

A cornerstone of HIV prevention clinical trials is providing a combination prevention package to all trial participants. The elements included in that standard of care (SoC) package evolve as new prevention modalities are developed. Pre-exposure prophylaxis (PrEP) was recommended by the World Health Organization for persons at high risk of acquiring HIV, but not all countries immediately adopted those recommendations. The South African Medical Research Council (SAMRC) convened a summit to discuss issues relating to SoC and PrEP in HIV prevention clinical trials taking place in lower- to middle-income countries (LMIC). Policymakers, regulators, ethicists, experts in law, researchers, representatives of advocacy groups, and the HIV Vaccine Trials Network (HVTN) presented a framework within which SoC principles could be articulated. A group of subject matter experts presented on the regulatory, ethical, scientific, and historic framework of SoC in clinical trials, focusing on PrEP in South Africa. Summit participants discussed how and when to include new HIV treatment and prevention practices into existing clinical guidelines and trial protocols, as well as the opportunities for and challenges to scaling up interventions. The summit addressed challenges to PrEP provision, such as inconsistent efficacy amongst different populations and various biological, virological, and immunological explanations for this heterogeneity. Advocates and community members propagated the urgent need for accessible interventions that could avert HIV infection. The meeting recommended supporting access to PrEP in HIV prevention trials by (1) developing PrEP access plans for HIV vaccine trials, (2) creating a PrEP fund that would supply PrEP to sites conducting HIV prevention trials via a central procurement mechanism, and (3) supporting the safety monitoring of PrEP. This report summarizes the presentations and discussions from the summit in order to highlight the importance of SoC in HIV prevention clinical trials.

**Supplementary Information:**

The online version contains supplementary material available at 10.1186/s13063-021-05754-z.

## Introduction

HIV prevention has evolved tremendously over the last decade. Traditionally, antiretroviral therapy (ART) was prescribed to people living with HIV (PLWH) to slow the progression of AIDS when CD4+ T-cell counts dipped below 200 cells per microliter. As ART reduces viral load, it was hypothesized to work as ‘treatment as prevention’ (TasP) to inhibit sexual transmission. In a trial of serodiscordant couples that compared early ART administration (irrespective of CD4 counts) to delayed, the early treatment reduced the risk of sexual HIV transmission to their uninfected partner by over 90% [1, 2]. This study, conducted by the HIV Prevention Trials Network (HPTN) HPTN 052, has since been joined by other trials that have shown undetectable viral loads prevent transmission and solidified the use of ART for TasP [3, 4].

The ‘inverse’ of HPTN 052 was investigating the efficacy of ART when administered to the HIV-uninfected partner in a serodiscordant relationship, a concept of pre-exposure prophylaxis (PrEP). Several randomized controlled trials (RCTs) comparing different combinations of oral tenofovir disoproxil fumarate (TDF) and TDF/emtricitabine (TDF/FTC, brand name Truvada) in serodiscordant couples have shown that PrEP can reduce HIV acquisition, although to different extents based on the population [5–7]. Prevention efficacy is robust in men, but contradictory results have been reported in women [5–9].

A cornerstone of HIV prevention clinical trials is providing a combination prevention package (e.g. risk reduction counselling, free condoms, diagnosis, and treatment for STIs) to all trial participants. The package provision is part of an obligation to minimize the participants’ risk of acquiring HIV, as assumption of risk can be underestimated in RCT participants [10, 11]. As new prevention modalities emerge, the prevention packages are adapted accordingly to provide the best care to participants whilst considering cost, population impact/efficacy, government and other funders’ roles, and clinical science integrity. In 2010, after iPrEx results showed PrEP efficacy in men who have sex with men (MSM) and transgender women, the HIV Vaccine Trials Network (HVTN) held a series of consultations with advocates, ethicists, and other stakeholders; surveys of participants; and discussions with protocol team leadership and research site investigators to evaluate the pros and cons of potential approaches to PrEP access in an ongoing HIV vaccine efficacy trial (HVTN 505) [12]. The group considered three options (ranging from providing information on PrEP to providing PrEP itself) and four main issues: researcher obligation to participants and their communities, effects on study design, health policy recommendations, and stakeholder opinions. The consensus was to provide PrEP information and referrals for PrEP access. For the latter, Gilead donated Truvada for any interested participant and the HVTN coordinated a contract with a mail-order pharmacy to ease access. As HVTN 505 was conducted only in the United States (US), ethical issues about differential health resources and regulatory standards in other countries were not addressed. Following this prevention package amendment, in 2012, PrEP was approved for use in populations at high risk of HIV infection in the US by the Food and Drug Administration [13].

In 2015, the World Health Organization (WHO) made a recommendation for the inclusion of oral PrEP as part of a combination HIV prevention package for people at substantial risk of HIV infection [14]. In 2017, TDF/FTC was licensed for use as PrEP in 17 countries and was included in the WHO Essential Medicines List (EML). At that time, although PrEP was approved in South Africa, its availability was limited to the National Department of Health (NDoH)-sanctioned sub-populations (specifically MSM and sex workers) and research-led demonstration projects. Unfortunately, this precluded heterosexual girls and women access to PrEP. Due to the high prevalence of HIV in women in South Africa, up to 24% in some areas [15], this population makes up a large proportion of participants in HIV prevention clinical trials conducted there. A substantial gap existed between participants in HIV prevention trials and local access to comprehensive prevention packages. The South African Medical Research Council (SAMRC) has a mandate to address issues that could potentially impact the conduct of health research, including clinical trials. As such, the SAMRC together with the HVTN convened a summit in Cape Town in 2017 to address PrEP access as a case study on the standard of care (SoC) in clinical trials in lower- and middle-income countries (LMIC). Whilst the summit focused on PrEP provision in South Africa, the issues raised are applicable to other forms of SoC across LMICs. This article summarizes the viewpoints and discussions that took place in November 2017 at the summit.

## Methods

As a result of the lack of PrEP licensure for those at high risk of HIV acquisition in South Africa, the SAMRC reached out to stakeholders in communities, government, academia, and pharmaceutical companies to discuss issues concerning PrEP provision as SoC in HIV prevention trials. The 2-day summit began with presentations from experts on ethics, regulatory processes, clinical studies, basic science, and community engagement, followed by a day of panel discussions with the goal of providing a comprehensive and thought-out plan on PrEP provision for HIV prevention trials in-country. The overall objective of the meeting was to engage regulatory, legal, and ethical frameworks, whilst considering perspectives of government, community organizations and advocates, and the funders of research to review the principles behind setting SoC for prevention clinical trials in LMIC to address the PrEP gap. There were 18 presentations on the first day (Supplemental Table 1) and three panel discussions on day 2. Both days were recorded and transcribed. This report summarizes the main themes from the summit, which resulted in a recommendation and Executive Statement from the SAMRC [16]. An Epilogue provides an update of what has happened in this space since the summit took place in 2017.

## Objectives:

- To outline what a SoC is and whether PrEP falls into this category for HIV prevention clinical trials in South Africa
- To present context of PrEP efficacy in South African females
- To discuss the approaches of how to provide PrEP as a SoC in HIV prevention trials in South Africa

## Themes of the summit

### Use of local and international guidelines to define SoC in efficacy trials

A modern definition of SoC is ‘that which a minimally competent physician in the same field would do under similar circumstances’ [17]. In the context of HIV vaccine RCT, SoC includes a basic prevention package that every participant has access to regardless of whether they receive vaccine or placebo. Local regulatory bodies ensure clinical trials include SoC and adhere to guidelines such as Sections 21 and 19 of the Medicines and Related Substances Act and General Regulations [18], the Nuremberg Code, Declaration of Helsinki and the Belmont Report [19–21], and the International Council for Harmonisation of Technical Requirements for Pharmaceuticals for Human Use (ICH) on Good Clinical Practice (GCP) [22, 23]. GCP requires clinical trial SoC to include informed consent, information about adverse outcomes, potential for post-trial treatment, and adverse drug event reporting. The South African Bill of Rights discusses the right to health in Section 27 of the Constitution: ‘The state has to take reasonable legislative measures within its available resources.’ The WHO, US Centers for Disease Control and Prevention (CDC), South African NDoH, and South African HIV Clinicians Society all have HIV prevention/treatment trial guidelines available. The purpose of these international guidelines on SoC is to promote consistency and ensure equity in clinical trials globally.

However, the definition of SoC can vary depending on context or location. PrEP licensure for ‘those at risk of HIV’ is such an example. Apart from a universal SoC, an international consensus standard considers SoC factors such as cost, the balance between desirable and undesirable effects, and acceptability to those who will receive treatment [24–26]. The SAMRC has funded the South African Guidelines Excellence (SAGE) project, which is a multi-partner, 5-goal project to develop and implement primary care guidelines in South Africa [27]. SAGE applies stakeholder expertise to efficiently identify and address resource limitations in SoC across the diverse health care system of South Africa. The SAGE project is in the midst of evaluating PrEP use for women in South Africa.

In 1996, the NDoH implemented the South African Standard Treatment Guidelines and Essential Medicines List (STG/EML), which determines access to and availability of medicines in the public sector, to ensure SoC for its citizens. Four criteria must be met for a medication to be approved to the EML: (1) public health need; (2) safety, efficacy, and quality; (3) pharma-economics/cost; and (4) practice considerations. In 2017, PrEP was not part of the EML although 26 countries had adopted the WHO recommendations for its use [28].

### Historical precedent for implementing new SoC for HIV prevention in South Africa

South Africa has, in the past, adopted international SoC guidelines for the prevention of mother-to-child transmission (PMTCT) of HIV. In 2002, the non-governmental organization Treatment Action Campaign (TAC) filed a lawsuit against the South African government over its limited ‘duty of care’ distribution of the antiretroviral drug nevirapine [29]. The government at the time denied scientific evidence surrounding HIV/AIDS and the Ministry of Health was opposed to any form of nevirapine rollout. TAC argued these limitations put PLWH and their newborns at a substantial health risk, violating their constitutional rights. The court ruled in favour of TAC that the government’s limited supply was unconstitutional as it denied nevirapine administration in the public health system where there was capacity and its use was medically indicated [30, 31].

In 2013, the Joint United Nations Programme on HIV/AIDS (UNAIDS) developed a new SoC for PMTCT of HIV. The new SoC, called Option B+, was to replace the previously used Options A and B that involved different ART regimens [32]. There was a large local debate after the international community proposed that South Africa implement Option B+, as the country was doing reasonably well with Options A/B. As it turned out, it was the right decision to move to Option B+; South Africa lowered the MTCT rate from 8% in 2009 to 2% in 2015 [33]. South African researchers used local data to make decisions based on the international guidance. This exemplifies how global decision-making can affect local SoC guidelines in South Africa.

### Implications of conflicting PrEP efficacy data for South African women

As stated before, SoC can vary based on context or region. The economic strength/status and medical infrastructure of a nation, city, or village can profoundly impact the type of health care its citizens receive. South Africa is one of the most unequal societies in the world, with a Gini coefficient of 0.63 [34] and an annual health expenditure of 8.2% gross national product [35]. There were an estimated 7.7 million PLWH in 2017, equating to an overall prevalence of ~ 14% [15]. However, heterosexual females are one of the most at-risk groups with an estimated prevalence of 24%. This is in contrast to, for example, the US where MSM and transgender women are at the highest risk (~ 12% prevalence) [36, 37].

In fact, the discrepancy in PrEP efficacy between MSM and heterosexual women is a major factor in its omission from the South African EML. A systematic review of oral PrEP RCT across a range of populations and settings concluded that it was effective in reducing HIV infection risk across gender, PrEP regimen, dosing, and mode of acquisition [38], and numerous studies have shown high efficacy of oral PrEP in MSM populations [6, 39–41]. Table 1 outlines the five efficacy trials of oral PrEP that enrolled women to date: Partners PrEP, Bangkok TDF, FEM-PrEP, VOICE, and TDF2. As depicted in the table, efficacy in heterosexual women is inconsistent. Numerous confounders have been suggested for the discrepancy, including biological factors (e.g. viral subtype, vaginal microenvironment) and behavioural factors (e.g. drug adherence, relationship status). In fact, a meta-analysis of these trials found that adherence to PrEP correlated with efficacy [43], and unfortunately, adherence has been lower for women in LMIC than MSM in high-income countries. Further data will be required to refine the estimates of PrEP efficacy in southern African women to aid in appropriate messaging, characterizing the impact of PrEP at a population level and within HIV prevention efficacy trials and understanding conditions that might compromise efficacy [43]. Despite these unknowns, the South African AIDS Committee guidelines included the provision of daily PrEP for high-risk populations in 2016 [44].

**Table 1:** Oral PrEP efficacy in women in 5 efficacy trials

Trial	PrEP efficacy (in women)	Ref
Partners PrEP		
TDF-FTC	66% (*p* = 0.005)	[5]
TDF	71% (*p* = 0.002)	[5]
Bangkok TDF	79% (*p* = 0.03)	[42]
FEM-PrEP	None	[9]
VOICE		
TDF-FTC	None	[8]
TDF	None	[8]
TDF2	None	[7]

Ethics committees responsible for evaluating SoC in clinical trials face a dilemma when there is a lack of consensus in the scientific community. In 2005, a prominent South African ethicist encouraged researchers that, ‘Contributing to sustainable improvements in health by progressively ratcheting the SoC upwards for research participants and their communities is an ethical obligation of those resource-rich countries who sponsor and implement research in poorer ones’ [45]. In 2016, the US-based ethicist Jeremy Sugarman referred to what is now highly quoted as the ‘rebuttable presumption’ [46]. According to Sugarman, the onus was placed on researchers to justify *not* offering PrEP to participants in HIV prevention trials. But, as stated before, this is not a simple issue when local governments do not provide that SoC to everyone in the target community.

### The National PrEP Technical Working Group and PrEP availability in South Africa

In October 2015, the NDoH convened a meeting to consider the soon-to-be-released WHO guidelines recommending PrEP use for all populations at substantial risk of HIV. At that meeting, the National PrEP Technical Working Group was formed, which has been an important vehicle for guiding PrEP introduction into South Africa. Shortly thereafter, the Medicines Control Council licensed TDF/FTC for PrEP use and, by June 2016, the beginnings of a publicly funded programme began where PrEP was provided to sex workers as part of the National Sex Worker Program. It was one of the first nationally funded PrEP programmes in Africa and was lauded by UNAIDS [47]. Between 2016 and 2017, publicly funded programmes provided nearly 3000 PrEP initiations at 17 sites across South Africa. A key take-home from the National Sex Worker Program data was that initially uptake was quite low, partly because prior to licensure, sex workers were sceptical as to the motives of including them in a programme. But with increasing awareness of PrEP and perhaps expansion of access to other populations, hesitancy seemed to decrease.

Since the kick-off of the National Sex Worker Program, the National PrEP Technical Working Group began implementing demonstration projects for adolescent girls and young women. Those projects have evaluated a number of strategies, including sex-positive materials targeted at young people, fixed facilities and mobile delivery models, drug-level feedback counselling, use of social clubs, and integration with services such as gender-based violence prevention. The demonstration projects have indicated that PrEP uptake, which varies across regions from 36 to 98%, increases when it is part of a broader prevention package that includes peer support, mobile services, and convenient operating times, or as part of a sexual reproductive health package. The working group has also investigated ways to reduce the burden of repeated visits for PrEP, particularly because the goal is to provide a convenient service for healthy populations. Finally, it is critical to train health care workers and ensure their positive attitudes in prescribing PrEP.

It is predicted that PrEP uptake will be variable and likely evolve over time. Clinical trialists should agree on an adequate package for adherence support and recognize that patterns of PrEP use will most likely vary over the course of a trial.

### Designing clinical trials in the era of PrEP

Offering PrEP to vaccine or placebo recipients in an HIV vaccine clinical trial will not truly disturb the ability to answer the question at hand: whether the new vaccine prevents HIV acquisition. This is true for most HIV vaccine studies, as the two modalities typically have different mechanisms of action. It would, however, have an impact. If participants use PrEP effectively, HIV incidence would decrease and therefore affect the statistical power of the primary objective (i.e. vaccine efficacy). For example, the HIV vaccine trial HVTN 505 increased its sample size from 1350 to 2500 participants to accommodate for PrEP use [48]. As such, consideration of PrEP provision must be addressed and analysed by statisticians early during the trial design process so that the scientific validity of the study is not compromised.

Another issue to consider is how PrEP provision is paid for. Most HIV prevention trials conducted in South Africa are sponsored by the US government (via the NIH), which stipulates research funds cannot be used for medication and SoC procedures, including lab work. The HVTN has previously raised philanthropic dollars to provide TDF/FTC through an online pharmacy when a participant received a prescription from their physician. This preserved community equity as well as lessened the potential burden on CRS staff of medical care that could detract from the necessary documentation and effort required to perform the trial. Typical vaccine efficacy trials last 5 years, and because NIH-sponsored trials are reliant on governmental budgeting, there is never a guarantee that grant funding will continue. In this system, an organization such as the HVTN can only guarantee PrEP access for the life of the trial. Ideally, local governments in the region a trial is conducted will step up at that point to provide post-trial access.

Representatives from pharmaceutical companies sponsoring clinical trials have acknowledged the importance of providing post-trial access to interventions that work. As these companies are themselves benefitting from the participants in a trial, the argument could be made that the companies owe the participants. There is precedent for including language in trial protocols that the sponsor will continue to provide access to a medication until it is accessible to study participants elsewhere. This post-trial access, some argue, is the industry’s responsibility. As PrEP could offer a population-level reduction in HIV prevalence, post-trial access would also provide benefit to local communities where the trials are conducted.

### Community voices should be considered when implementing PrEP access in clinical trials

The benefits of community engagement and collaboration between clinical trial networks and community advocates are well documented [49–51]. Community Advisory Board (CAB) members are integral colleagues in the operations of trials conducted by the US NIH-funded HIV/AIDS networks, such as the HVTN. CABs help ensure that SoC given to clinical trial participants are ethical, scientifically valid, and developed with sincere collaboration with communities and advocates. In regard to HIV prevention trials conducted in South Africa, community stakeholder involvement in decision-making on PrEP provision is essential to ensuring protocols are acceptable to trial participants. As such, advocates and community representatives should be engaged in the design of the PrEP plans. Communities should feel ‘ownership’ rather than ‘buy-in.’ It is also important there be a mechanism for advocates and community members to monitor and inform SoC evolution so that the care is accountable, transparent, and client centred.

As there are approximately 2000 new HIV infections in young women in South Africa each week, it is imperative that if an effective agent against HIV acquisition exists, its use should not be put on hold. Clinical trial participants will need choices for HIV prevention; a one-size-fits-all approach will not likely make a significant dent in acquisition rates. PrEP should be provided to participants who choose to take it either through easily accessible health care organizations/clinics in the region or by the clinical trial sites themselves. In addition, the success of PEPFAR in ART provision in Africa can be viewed as a model for government action to drastically increase access to PrEP [52].

## Conclusions

The topic of how to use PrEP ethically, safely, and effectively has been a key consideration in the planning of prevention trials planned for South Africa. The recommendations of the Ministry of Health, South African ethics boards, research teams, and the vulnerable communities should be heeded in clinical trials conducted in any LMIC. Consensus across all these stakeholders is crucial for deciding how and when to provide access to modalities that have not yet fully bloomed in-country.

During the SoC summit discussions, it became clear that HIV prevention researchers should move towards making PrEP available as part of the HIV prevention package for study participants of a clinical trial. South African investigators and collaborators, including the NIH’s two largest clinical trial networks working in South Africa (HVTN and HPTN), proposed to work together as standard of prevention services evolve in southern Africa. The scientific validity of a clinical trial must be considered in decisions made regarding PrEP access. In addition, it is crucial that investigators engage their government bodies or working groups to motivate the support of PrEP demonstration projects close to research sites, not only for PrEP access, but to also provide HIV testing and PrEP-related safety monitoring.

This summit demonstrated that considerations of SoC in a clinical trial setting are complex. These complexities are dynamic and contextual nuances may vary from site to site. Both local and international guidelines can inform the review and the need to modify the standard of prevention to ensure LMIC clinical trials are performed with the highest ethical standards.

## Epilogue

Following the summit, the SAMRC, with additional support from the Fred Hutchinson Cancer Research Center (parent institution of the HVTN) and industry partners, set up a PrEP fund for drug acquisition and laboratory monitoring for HIV prevention study participants across sub-Saharan Africa. This enabled the SAMRC to purchase PrEP at state tender prices and utilize pre-existing negotiated laboratory contracts and within-study systems to extend available PrEP funds. PrEP drug provision and related essential laboratory monitoring support were subsequently initiated for interested and eligible HVTN efficacy trial participants at the 24 clinical trial sites conducting trials in sub-Saharan Africa (HVTN 702 [ClinicalTrials.gov: NCT02968849), HVTN 703/HPTN 081 [ClinicalTrials.gov: NCT02568215], and HVTN 705/HPX2008 [ClinicalTrials.gov: NCT03060629]). PrEP was offered at each instance of risk reduction counselling along with other prevention methods provided during participant study visits and during the informed consent process. Where possible, sites could offer PrEP from their own pharmacies and staff were trained to prescribe and manage PrEP initiation and follow-up. Each clinical site received an up-to-date list of pharmacies and clinics that stocked and prescribed PrEP in their areas. PrEP training for site staff was offered periodically to enable PrEP prescribing and management. The more recent HPX3002/HVTN 706 (Mosaico) trial conducted amongst MSM and transgender individuals in the Americas and Europe offered a slightly different approach to PrEP administration to ensure all participants could receive access to the highest standard of prevention. The trial was designed to give volunteers three options regarding PrEP: (1) participants already on PrEP or who wished to take PrEP will not be included in the study, but referred to PrEP resources in their community if requested; (2) participants who decide to take PrEP after receiving vaccination can remain in the study; and (3) participants not wanting to take PrEP can be included in the study [53].

## Supplementary Information

**Additional file 1: Supplement Table 1.** Summit presentations.

## Data Availability

N/A

## Abbreviations

SAMRC: South African Medical Research Council
SoC: Standard of care
LMIC: Lower- and middle-income countries
WHO: World Health Organization
TDF/FTC: Tenofovir disoproxil fumarate/emtricitabine
EML: Essential Medicines List
PrEP: Pre-exposure prophylaxis
NDoH: National Department of Health
MSM: Men who have sex with men
HVTN: HIV Vaccine Trials Network
ICH: International Council for Harmonisation of Technical Requirements for Pharmaceuticals for Human Use
GCP: Good Clinical Practice
RCT: Randomized controlled trial
PMTCT: Prevention of mother-to-child transmission
ART: Antiretroviral therapy
CAB: Community Advisory Board
