# Supplementary material for: Meeting report: South African Medical Research Council Standard of Care in Clinical Research in Low- And Middle-Income Settings Summit, November 2017
Source: Trials. 2021 Nov 6;22:778. doi: 10.1186/s13063-021-05754-z (PMC8572437; doi:10.1186/s13063-021-05754-z)
Supplement: Supplementary file 1 — Additional file 1: Supplement Table 1. Summit presentations. [file 13063_2021_5754_MOESM1_ESM.docx]

**Supplement Table 1. Summit presentations.**

| DAY 1: THURSDAY, 02 NOVEMBER 2017 |
| --- |
| REGULATORY CONSIDERATIONS FOR STANDARD OF CARE IN CLINICAL RESEARCH |
| Regulatory consideration for standard of care in treatment and prevention |
| Current STGs as the minimum standard of care in trials |
| Integrating Helsinki and other ethical guidelines in South Africa for clinical research |
| Standard of care in cancer studies in South Africa |
| STANDARD OF CARE CONSIDERATIONS IN LMICS AND SA |
| Factors to be considered in implementing standard of care in South Africa – case studies of Treatment as Prevention, PMTCT, PrEP, medical male circumcision |
| Ethics and law considerations for standard of care – case study of PrEP |
| Standard of care issues in Zimbabwe |
| Standard of care considerations in LMIC |
| Evidence and guideline development (SAGE) |
| BIOLOGICAL AND ADHERENCE CONSIDERATIONS |
| Biological factors that affect Tenofovir in the vagina |
| Experience with PrEP in clinical trials – vaginal microbiome |
| Update on demonstration projects in SA – challenges and successes |
| What mechanisms exist for participants to access PrEP at demonstration sites |
| Deliberations from Community Stakeholder Engagement |
| PREP CONSIDERATIONS IN THE DESIGN OF HIV PREVENTION TRIALS |
| Efficacy data of PrEP in women |
| Meta-analysis of PrEP – use of evidence in policy |
| ECHO study |
| 083/084 study design – regulatory and statistical issues |
| DAY 2: FRIDAY, 03 NOVEMBER 2017 |
| PANEL DISCUSSIONS |
| Sponsor’s and implementer’s role in providing standard of care |
| Regulatory/ethics/legal considerations in establishing standard of care in clinical trials |
| Shared views from advocacy and community lens |
|  |
